# Socioeconomic and geographical inequalities in using skilled birth attendants during delivery in Bangladesh over two decades

**DOI:** 10.1186/s12884-023-05754-8

**Published:** 2023-06-09

**Authors:** Syed Sharaf Ahmed Chowdhury, Satyajit Kundu, Azaz Bin Sharif

**Affiliations:** 1 Department of Public Health, North South University, Dhaka, 1229 Bangladesh; 2 Global Health Institute, North South University, Dhaka, 1229 Bangladesh; 3 Faculty of Nutrition and Food Science, Patuakhali Science and Technology University, Patuakhali, 8602 Bangladesh

**Keywords:** Skilled birth attendant, Maternal health, Inequalities, Bangladesh, BDHS

## Abstract

**Background:**

Maternal and neonatal mortality is a major public health concern globally. Evidence supports that skilled birth attendants (SBA) can significantly reduce maternal and neonatal mortality. Despite the improvement in SBA use, Bangladesh lacks evidence of equality in SBA use across socioeconomic and geographic regions. Therefore, we aim to estimate the trends and magnitude of inequality in SBA use in Bangladesh over the last two decades.

**Methods:**

Data from the last 5 rounds of Bangladesh Demographic and Health Surveys (BDHS; 2017-18, 2014, 2011, 2007, and 2004) were used to measure the inequalities in the SBA use utilizing the WHO’s Health Equity Assessment Toolkit (HEAT) software. Inequality was assessed by four summary measures, namely, Population Attributable Risk (PAR), Population Attributable Fraction (PAF), Difference (D), and Ratio (R) based on the four equity dimensions: wealth status, education level, place of residence, and subnational regions (divisions). Point estimates and a 95% confidence interval (CI) were reported for each measure.

**Results:**

An increasing trend in the overall prevalence of SBA use was observed (From 15.6% in 2004 to 52.9% in 2017). We found significant inequalities in SBA use in every wave of BDHS (from 2004 to 2017), with the result concentrating on the rich (in 2017, PAF: 57.1; 95% CI: 52.5–61.7), educated (in 2017, PAR: 9.9; 95% CI: 5.2–14.5),  and people from urban areas (in 2017, PAF: 28.0; 95% CI: 26.4–29.5). We also identified geographic disparities in SBA use favoring Khulna and Dhaka divisions (in 2017, PAR: 10.2; 95% CI: 5.7–14.7). Our study also observed inequality in using SBA among Bangladeshi women decreased over time.

**Conclusion:**

To increase SBA use and to decrease inequality in all four equity dimensions, disadvantaged sub-groups should be prioritized in policies and planning for program implementation.

## Introduction

Maternal and child mortality has been a public health concern for many years. The reduction of maternal and child mortality is considered one of the main indicators to be achieved under goal 3 of the sustainable development goals (SDGs). Despite significant improvement, globally, the maternal mortality ratio (MMR) is still 223 deaths per 100,000 live births which is unacceptably high and far from the fulfillment of the SDGs target of 70 deaths per 100,000 live births [1]. The MMR is the highest in low- and middle-income countries and accounts for about 95% of maternal deaths worldwide. In comparison, South Asia alone contributed about 16% of global maternal death in 2020 [2]. Most causes associated with maternal mortality are preventable if care is provided by skilled professionals trained to address complications and manage them properly [3].

The World Health Organization (WHO), jointly with International Confederation of Midwives (ICM) and International Federation of Gynecology and Obstetrics (FIGO), clearly states that a skilled birth attendant is a health professional who is accredited, educated, and trained to develop a set of skills to manage uncomplicated pregnancy and childbirth and identify complicated pregnancies, manage if possible, and when required refer mother and the newborn to appropriate care facilities [4]. Skilled birth attendance is critical for maternal and child health improvement [4, 5]. Studies have shown that skilled birth attendance is associated with a reduction in maternal mortality [6]. Following the evidence, SBA coverage has increased significantly globally, with around 80% of the birth coverage [7].

According to Save the Children and UNFPA, attendance of childbirth by a supported and trained skilled birth attendant can reduce 43% of the neonatal mortality [8] and two-thirds of the maternal mortality [9] which in numbers will add up to 3.6 million lives saved at the end of the year by doubling the number of SBA according to another report by UNFPA in 2011 [10]. Despite glowing evidence and enormous success in reducing maternal mortality through the supervision of skilled birth attendance, low and middle-income countries like Sub-Saharan Africa and South Asia have a much lower prevalence of SBA attendance compared to European countries [11–13]. Although over the years, the prevalence of SBA has improved globally [14], different studies conducted in Ethiopia [15], Niger [16], Mali [16], and Sierra Leone [16] identified huge socioeconomic and geographical variations in the prevalence of SBA utilization. For example, studies conducted in Bangladesh [17, 18], Ghana [19, 20], and Ethiopia [21, 22] reported higher use of SBA among the people in the higher economic quintile, those attaining higher education, and women with a friend using SBA compared to the women who are at odds. The overall increase in the prevalence of SBA use may not be enough to reduce maternal mortality uniformly unless the inequality in SBA use is addressed.

In the last 10 years, Bangladesh has notablyy increased the prevalence of SBA deliveries. From 21% to 2007, the prevalence of SBA deliveries increased to 53%, according to the latest Bangladesh Demographic and Health Survey (BDHS) 2017-18 [23]. This increased prevalence is still lower than the target by Bangladesh’s fourth Health Population and Nutrition Sector Development Program, which aimed to achieve the SBA use of 65% by 2022 [23]. Unavailability of the SBA due to the dysfunctionality of the health system in SBA distribution, urban preference tendency among the SBAs, and cultural beliefs of male or senior family members making the health decision for women in Bangladesh might influence the SBA use during delivery and contribute to SBA-related inequality. Previous research using 2014 BDHS data identified significant geographical disparity in the use of SBA among women of the Khulna and Sylhet divisions compared to the other administrative divisions [24]. In addition, a significant rural-urban variation in the prevalence of SBA was also evident from a previous Bangladeshi study [23]. Considering the findings of previous literature from Bangladesh and other developing countries, we hypothesized that there might be socioeconomic and geographical disparities in SBA use during delivery among Bangladeshi women.

Different factors have been found to be associated with utilizing the SBA during delivery. Factors like education, wealth quintile, place of residence, and sub-national region were commonly found to be significantly associated with SBA use, which is evidenced by the previous literature from Bangladesh [17, 18, 23], Nepal [25], Tanzania [26], and Ethiopia [27]. Several other factors likethe number of ANC visits during pregnancy [26, 27], the distance of the health facility from the place of residence [25], were also reported to be associated with the utilization of SBA. Moreover, knowledge about the danger signs of pregnancy [25], number of children [26], media exposure [27], and birth order [23, 27] were also found to be reported as the associated factor of SBA use during delivery in different occasions.

Though several studies [17, 18] have been conducted with the aim to determine the factors influencing the use of SBA during childbirth at a certain point in time, to our knowledge, no studies have been conducted that pointed out the inequality of SBA use in Bangladesh over time determining the trend and magnitude of inequality. Hence, this study aims to estimate the trends and magnitude of socioeconomic and geographical inequalities of SBA use in Bangladesh through different equity dimensions over two decades using data from five previous BDHS. The finding from this study will help the policymakers to address the inequality in different dimensions of SBA use and motivate the researcher to conduct further research.

## Methods

### Study design and sampling

Five rounds of BDHS data from 2004, followed by 2007, 2011, 2014, and 2017-18 were analyzed to measure the inequality of SBA use. BDHS is a nationally representative cross-sectional survey which is conducted by the National Institute of Population Research and Training (NIPORT) and the Ministry of Health and Family Welfare of Bangladesh in partnership with USAID as a part of the MEASURE program of DHS conducted in low and middle-income countries and all their data are stored in the WHO’s Health Equity Assessment Toolkit (HEAT). BDHS uses two-stage stratified sampling technique to collect data, where enumeration areas (EAs) were selected across the nation in the first stage, which were also considered the primary sampling units (PSUs). In the second stage, households were selected from each PSU. The detailed methodology, including the survey’s sampling techniques, is described in the final report of the latest BDHS [23].

### Outcome variable

The use of SBA by Bangladeshi women was the primary outcome variable of this study. SBA was defined as when the births were assisted by the skilled personnel who, according to the BDHS, are the qualified doctors, nurses, midwives, or paramedics; family welfare visitors (FWVs); community skilled birth attendants (CSBAs); and sub-assistant community medical officers (SACMOs) [23]. We considered the SBA use among Bangladeshi women in the two or three years preceding the surveys [23]. Women were considered to use SBA if their delivery was attended to or assisted by skilled health personnel [4]. The response for the outcome variable was dichotomous (coded as 1 for SBA use and 0 for unskilled attendant) [28].

#### Equity dimensions

The inequality for the use of SBA was measured based on 4 equity dimensions. The dimensions were economic status, education, place of residence, and sub-national region. The economic status (household wealth quintiles) was constructed using the Principal Component Analysis (PCA) based on the household income, assets, and characteristics that were then ranked into poorest, poorer, middle, richer, and richest sub-groups [29]. Women’s level of education was categorized as no education, primary, and secondary/higher. The place of residence of the respondents was classified as urban and rural, and administrative divisions of Bangladesh were considered as the sub-national regions for measuring inequality.

### Statistical analysis

For analyzing the inequality of the SBA use, we used the Health Equity Assessment Toolkit software (version 4.0) by WHO [30]. Data analysis using this software was carried out in two steps. In the first stage, data were disaggregated by the four equity dimensions (economic status, education of women, place of residence, and sub-national region) to estimate and distribute the prevalence of SBA use across the dimensions. The inequality was measured in the second stage, where both absolute and relative summary measures were calculated. We calculated Difference (D) and Population Attributable Risk (PAR) for the absolute measure. Population Attributable Fraction (PAF), and Ratio (R) were estimated as relative measures. Evidence showed that absolute and relative measures are important to be reported in a single study measuring health inequality [30, 31]. These 4 measures were calculated for each equity dimension in which D and R were considered simple measures,simple measures, and the PAF and PAR were complex measures (weighted measurement). In the case of the binary or non-ordered dimensions like the place of residence or geographical regions, the simple measure D is calculated simply by subtracting the lowest estimate (rural) from the highest estimate (urban), and R is the quotient obtained by dividing the group with the highest prevalence (Khulna) by the group with lowest prevalence (Sylhet). On the other hand, for the ordered dimension like wealth quintile and education level, D is the result of the subtraction between the most advantageous (richest) and most disadvantageous group (poorest), and R is the ratio between the advantageous (secondary or higher education) and disadvantageous group (no education) [32]. To measure the complex measures like PAR and PAF, the national average was considered as the population average. PAR is calculated as the difference between the group with the highest estimate (Y_highest_) and the national average ($\mu )$ irrespective of the indicator type i.e., PAR = Y_highest_ – $\mu$. The PAF is product of the result obtained by dividing the PAR by the national average and 100 i.e., PAF = (PAR/$\mu$) × 100 [28, 33]. The detailed procedure and calculation of the summary measures are described elaborately elsewhere [30, 31].

The PAF and PAR values can be positive or negative in the calculation where the positive value is assumed to favor the health intervention, and the negative value indicates the adverse outcome [34]. Therefore, the higher value of PAF and PAR will indicate higher inequality regardless of the direction, and the inequality will be absent if the PAF and PAR values are 0. In contrast, inequality will be absent in the case of simple measures if the values of D and R, respectively become 0 and 1. We have calculated the point estimate of the measures and presented the result with a 95% confidence interval (CI). With all inequality measures, a confidence interval (CI) that did not include 0 was regarded as statistically significant; except for R, where the CI should not contain 1 to be considered statistically significant [34].

## Results

### Distribution of SBA use in different equity dimensions

The trend of SBA use among Bangladeshi women of different socioeconomic status is shown in Table 1 over the time from 2004 to 2017. An increased prevalence in SBA use was observed among all sub-groups, while women from richest quintile was found to be highest in SBA use in 2004 (45.8%), which raised to 83.1% in 2017. Similarly, an increasing pattern of SBA use is also found among women with higher education. For instance, in 2017, women who had secondary/higher education utilized SBA (62.7%) higher than the women who had no formal education (29.0%).

**Table 1:** Trends in the prevalence of skilled birth attendant, disintegrated across four inequality dimensions, from years 2004 to 2017

Inequality Dimension	2004 (15.6%)		2007 (20.9%)		2011 (31.7%)		2014 (42.1%)		2017 (52.9%)	
	n	Estimate (95% CI)	n	Estimate (95% CI)	n	Estimate (95% CI)	n	Estimate (95% CI)	n	Estimate (95% CI)
**Economic Status**										
Quintile 1 (poorest)	1029	3.7 (2.5–5.4)	758	6.8 (4.9–9.4)	1136	11.5 (9.3–14.1)	1084	17.9 (14.6–21.8)	1108	28.2 (24.7–32.1)
Quintile 2	831	4.4 (3.0-6.5)	779	7.3 (5.2–10.0)	1003	18.6 (15.4–22.2)	932	29.9 (25.7–34.5)	1106	40.2 (36.4–44.1)
Quintile 3	848	12 (9.7–14.9)	699	12.9 (10.1–16.3)	974	28.2 (24.9–31.8)	943	38.8 (32.1–45.9)	1020	52.8 (48.5–57.0)
Quintile 4	717	20.2 (16.2–24.9)	694	25.8 (21.7–30.3)	963	43.2 (39.5–46.9)	995	52.0 (47.4–56.5)	1071	62.4 (58.7–66.0)
Quintile 5 (richest)	695	45.8 (41.7–50.0)	659	56.4 (52.3–60.5)	881	63.8 (59.4–68.0)	950	74.4 (70.5–77.9)	1034	83.1 (80.3–85.5)
**Level of Education**										
No Education	1463	4.5 (3.4-6.0)	856	6.3 (4.5–8.8)	892	12.6 (10.1–15.7)	704	17.1 (13.2–21.9)	351	29.0 (23.8–35.0)
Primary School	1256	11.4 (9.5–13.6)	1103	9.9 (8.1–12.1)	1485	20.0 (17.3–23.0)	1380	29.5 (26.1–33.1)	1471	35.0 (31.4–38.8)
Secondary / Higher	1400	30.8 (28.1–33.7)	1619	36.1 (32.9–39.4)	2579	45.0 (42.3–47.6)	2820	54.4 (51.0-57.8)	3515	62.7 (60.4–65.0)
**Place of Residence**										
Rural	3307	11.0 (9.6–12.7)	2828	15.7 (13.6–18.2)	3835	25.2 (22.9–27.6)	3637	35.6 (32.3–39.1)	3911	47.5 (44.6–50.3)
Urban	813	33.9 (29.7–38.3)	761	40.0 (35.1–45.1)	1121	53.7 (49.3–58.1)	1267	60.5 (56.1–64.8)	1427	67.7 (63.8–71.3)
**Sub-National Regions**										
Barisal	237	14.3 (9.2–21.5)	219	14.7 (11.3–18.8)	273	28.4 (23.2–34.3)	279	36.7 (26.8–47.8)	303	46.9 (40.8–53.0)
Chattogram	920	12.7 (9.9–16.2)	798	20.9 (15.5–27.6)	1176	29.7 (25.0-34.9)	1075	43.9 (37.6–50.5)	1141	50.6 (44.6–56.7)
Dhaka	1250	18.5 (15.7–21.7)	1132	23.1 (19.3–27.4)	1510	31.5 (27.3–36.1)	1740	43.5 (37.3–49.8)	1359	60.5 (54.8–65.9)
Khulna	450	23.6 (19.3–28.4)	328	31.5 (25.2–38.4)	463	49.0 (43.4–54.5)	387	58.2 (51.8–64.4)	482	63.1 (57.9–68.0)
Mymenshingh	-	-	-	-	-	-	-	-	451	41.6 (36.5–46.9)
Rajshahi	896	11.8 (8.9–15.6)	792	18.1 (15.3–21.2)	646	30.9 (25.2–37.2)	488	41.6 (35.9–47.6)	622	55.4 (49.8–60.8)
Rangpur	-	-	-	-	513	28.7 (24.3–33.6)	461	37.9 (35.9–47.6)	555	49.2 (42.0-56.4)
Sylhet	366	12.7 (10.0-16.2)	319	13.2 (9.0-18.9)	375	24.4 (20.1–29.4)	474	27.1 (33.0-43.1)	426	40.4 (33.5–47.8)

CI: Confidence Interval, Mymensingh division was separated from Dhaka division in 2015, and Rangpur division was separated from Rajshahi division in 2010. Hence, the estimates for BDHS 2004–2014 data of Mymensingh, and BDHS 2004–2007 data of Rangpur division are not shown in the table

An uplifting trend for the overall prevalence of SBA was also found, where the prevalence of SBA was 15.6% in 2004, increased to 31.7% in 2011, and 52.9% in 2017. Furthermore, women from urban areas (67.7%) and belonging to the sub-national region of Khulna (63.1%) and Dhaka (60.5%) had the highest prevalence of SBA in 2017. The result also shows that Sylhet was found to have the lowest prevalence (12.7%) in 2004 and still lagged behind having the lowest prevalence of SBA among other sub-national regions in 2017 (40.4%) (Table 1).

### Magnitude of the inequalities in the SBA use

Both socioeconomic and geographical inequalities in the prevalence of SBA use were found based on all summary measures. Wealth-driven disparities were obtained by simple (D, R) and complex measures (PAF, PAR) with a higher concentration among the poorest sub-group than the Richest. For instance, the PAF measure 57.1 (95% CI: 52.5–61.7) in 2017 shows significant inequalities favoring those from the highest wealth quintile. Education-related inequality was found in all survey waves disfavoring the women with no education than those with secondary/higher education. For example, the absolute measure PAR (9.9; 95% CI: 5.2–14.5) and relative measure PAF (18.6; 95% CI: 9.8–27.4) in 2017 indicated significant inequalities with higher burden among women without formal education (Table 2).

**Table 2:** Inequality indices estimates of the factors associated with prevalence of women attended by a skilled birth attendant, years 2004–2017

Inequality Dimension	2004		2007		2011		2014		2017	
	Estimate	LB-UB	Estimate	LB-UB	Estimate	LB-UB	Estimate	LB-UB	Estimate	LB-UB
**Economic status**										
D	42.1	37.8–46.5	49.6	45.0-54.3	52.3	47.4–57.2	56.5	51.3–61.7	54.8	50.3–59.3
PAF	194.8	187.5-202.1	170.5	162.1-178.8	101.5	95.9–107.0	76.9	71.8–82.0	57.1	52.5–61.7
PAR	30.3	29.2–31.4	35.6	33.8–37.3	32.1	30.4–33.9	32.3	30.2–34.5	30.2	27.7–32.6
R	12.3	8.4–18.2	8.3	6.0-11.5	5.5	4.5–6.9	4.2	3.4–5.1	2.9	2.6–3.4
**Level of Education**										
D	26.3	23.3–29.4	29.8	25.9–33.6	32.3	28.5–36.1	37.3	31.8–42.8	33.7	27.6–39.7
PAF	98.3	91.8-104.8	72.7	65.1–80.2	42.0	35.4–48.6	29.4	23.0-35.8	18.6	9.8–27.4
PAR	15.3	14.3–16.3	15.2	13.6–16.8	13.3	11.2–15.4	12.4	9.7–15.1	9.9	5.2–14.5
R	6.8	5.1–9.1	5.7	4.1-8.0	3.6	2.8–4.5	3.2	2.5–4.1	2.2	1.8–2.6
**Place of Residence**										
D	22.8	18.3–27.4	24.3	18.8–29.8	28.5	23.5–33.5	24.9	19.4–30.5	20.2	15.5–24.9
PAF	117.8	113.5-122.2	91.7	87.8–95.6	69.7	67.2–72.2	43.9	41.9–46.0	28.0	26.4–29.5
PAR	18.3	17.6–19.0	19.1	18.3–19.9	22.1	21.3–22.9	18.5	17.6–19.3	14.8	14.0-15.6
R	3.1	2.5–3.7	2.5	2.1–3.1	2.1	1.9–2.4	1.7	1.5–1.9	1.4	1.3–1.5
**Sub-National Region**										
D	11.7	6.1–17.4	18.3	10.1–26.4	24.5	17.3–31.8	31.1	21.1–41.1	22.7	13.9–31.4
PAF	51.5	39.1–63.9	50.7	33.3–68.2	54.7	41.3–68.0	38.4	29.2–47.6	19.3	10.8–27.8
PAR	8.0	6.1–9.9	10.6	6.9–14.2	17.3	13.1–21.5	16.1	12.3–20.0	10.2	5.7–14.7
R	2.0	1.4–2.8	2.4	1.6–3.6	2.0	1.6–2.5	2.1	1.6–2.9	1.6	1.3–1.9

LB: Lower Bound of Confidence Interval, UB: Upper Bound of Confidence Interval, D: Difference, PAR: Population Attributable risk, PAF: Population Attributable Fraction, R: Ratio

We also found significant absolute and relative inequalities in the geographical (non-ordinal) dimensions like the place of residence and subnational region from 2004 to 2017. A rural-urban disparity was obtained over time, with women from the rural areas being the disfavored sub-group. For instance, PAF and PAR measures of 28 (95% CI: 26.4–29.5) and 14.8 (95% CI: 14-15.6) in 2017, respectively, describe significant pro-rich inequalities. Similarly, with a higher concentration of SBA use in the Khulna and Dhaka division, the regional inequalities were declining in the past two decades, disfavoring the Sylhet division. The significant regional inequalities were evident from the PAF of 19.3 (95% CI: 10.8–27.8) as the relative measure and D of 22.7 (95% CI: 13.9–31.4) as the absolute measure in 2017 ﻿(Table 2).

## Discussion

In this study, we have investigated the trend and inequalities in the prevalence of SBA use in Bangladesh over the last two decades, from 2004 to 2017, using the nationally representative BDHS data. It was observed that the use of SBA has increased from 15.6% to 2004 to 52.9% in 2017. This increasing pattern of SBA use among women is in line with the study conducted in Guinea [28], Ethiopia [15], and Ghana [35]. This finding could be due to the increased access to the number of health facilities during these two decades [36]. Another reason behind this study finding could be the increased number of skilled birth attendants due to the government initiatives in 2003. The initiative was to provide 2 trained community skilled birth attendants in each union, which resulted in increased SBA access over time [37]. Besides different non-government organizations like BRAC is also working on the capacity building of SBA in Bangladesh [38].

This study revealed that the prevalence of utilizing SBA was higher among the women from households with highest economic status than the women from lowest economic sub-group. This pattern was consistent over time and coincided with several previous studies [35, 39]. The prevalence of SBA use was also concentrated in the rich quintile in studies conducted in Bangladesh [17, 40]. Despite the increase in the prevalence in the SBA use among the women from all wealth quintiles over time, persistent inequality remains. This could be due to the much higher increase in SBA use among the women in the richest quintile compared to a slow rise in the poorest quintile. Besides, the cost of SBA use, which is certainly higher for the women from poorest quintile to manage [17]. And the SBA’s mostly prefer to attend the households that would pay them well [41]. Another reason might be that wealthier women tend to make their own decision regarding the health care, especially when it comes to the baby’s health [42].

Women with secondary or higher education were found to have higher prevalence of SBA use than those without formal education. This finding corroborates with the studies on the inequality in SBA use conducted in Guinea [28], Ghana [35], and Ethiopia [15]. Similar results were observed in the studies that looked at the factors determining the SBA use in Ghana [43], and Bangladesh [17, 18]. This result can be explained by the increase in awareness about health problems among educated women compared to non-educated women [44]. Educated women are also found to be more empowered and confident in taking their own healthcare decisions than those with no education [45, 46]. This could also affect their choice of SBA use during delivery. Besides, educated women are more aware of the availability and accessibility of healthcare services in their surroundings [44]. On the other hand, higher education is also associated with the wealth status. Since higher educated women are more likely to have better job they are found to be financially independent [17] and more likely to make choices like whether to use SBA during delivery. However, further qualitative exploration is warranted to know the in-depth scenario of why SBA utilization are lower among women with low socioeconomic status in Bangladesh.

Urban-rural disparities in the use of skilled birth attendants was observed in this study. SBA use was found to be higher among urban women than rural women, which is in line with the findings of previous studies done in Guinea [28], Bangladesh [17], India [47], and Ethiopia [48]. Lack of quality service in rural areas could be one of the factors behind the rural-urban disparities in the SBA use. This finding could also be explained by the lack of awareness among rural women compared to urban women, which is also associated with lower educational level and socioeconomic status [24, 41]. Another reason behind the less utilization of SBA may be the greater distance of health facilities from rural households [49]. The urban preference tendency (i.e., skilled professionals are more likely to work in the urban site due to better facility and financial benefits than the rural areas) of the SBAs and social norms in the rural community could be another plausible reason in the disparities in the SBA use among the women of urban and rural residence.

Regional inequality in the prevalence of SBA use was significant with the highest prevalence of SBA use in Khulna division, and the lowest in Sylhet division. results were found in studies conducted previously in Bangladesh using the BDHS data [17, 50]. Khulna was found to have a greater prevalence of women in the higher wealth quintile, attaining higher education, and more antenatal care visits than Sylhet [50]. These could be the reasons for the difference in the use of SBA, since all of these factors are associated with more SBA use [51, 52]. Again, the lack of awareness among the women in Sylhet, very little knowledge about the danger signs of pregnancy and child birth, and religious superstitions among many families were reported to be the reasons behind the low prevalence of SBA use in Sylhet and the lack of access to health care. Besides, not choosing skilled birth attendants for delivery was found to save them both money and travel from home, leading to a lower prevalence than in any other subnational region in Bangladesh [53].

### Strengths and limitations

In this study, the inequality measures were calculated by WHO HEAT software which increases the quality of the study due to the use of both absolute and relative summary measures. As we have used data from nationally representative surveys, the result of our study could be generalizable across the populations. We have also used simple and complex (weight was considered) measures that upheld the appropriateness of measuring the magnitude of inequalities. This study will be helpful for policymakers to innovate new program ideas to narrow down the inequalities in SBA use in both socioeconomic and geographical dimensions.

There are a few limitations in this study as well. Since we used the data from cross sectional studies, we could not show any causal effect for the inequality of SBA use among the women in different dimensions. Also, due to the choice of analytic strategy, we could not consider other dimensions of inequality, limiting us to measuring inequalities in different social, demographic, and cultural domains.

## Conclusion

From the results of this study, we conclude that, despite the increasing trend in the prevalence of SBA use and narrowing of the inequalities in the last two decades, there is significantly persistent inequalities exist between the advantaged group being the rich, educated, and urban resident and the disadvantaged group being the poor, non-educated and rural women. Besides, regional disparities were also observed, with a significant gap in prevalence of SBA use between the Khulna and Sylhet divisions. To curve the inequality in the SBA use among Bangladeshi women, dimension-based intervention designing is necessary by focusing on the disadvantaged sub-groups. Therefore, the government and policymakers should consider the planning and implementation of programs explicitly addressing the disadvantaged group to minimize inequality. Future studies may consider qualitative and prospective research to find the contextual differences and the causes of inequality in multiple dimensions.

## Data Availability

The study used data from the 2017–2018 Bangladesh Demographic and Health Survey. The data set is available at: https://dhsprogram.com/data/available-datasets.cfm.

## Abbreviations

SBA: Skilled Birth Attendant
HEAT: Health Equity Assessment Toolkit
BDHS: Bangladesh Demographic and Health Survey
SDG: Sustainable Development Goals
MMR: Maternal Mortality Ratio
WHO: World Health Organization
ICM: International Confederation of Midwives
FIGO: International Federation of Gynecology and Obstetrics
EA: Enumeration Area
PSU: Primary Sampling Unit
CI: Confidence Interval
PAF: Population Attributable Fraction
PAR: Population Attributable Risk
PCA: Principal Component Analysis
BRAC: Bangladesh Rural Advancement Committee
